# Supplementary material for: Entanglement and manipulation of the magnetic and spin–orbit order in multiferroic Rashba semiconductors
Source: Nat Commun. 2016 Oct 21;7:13071. doi: 10.1038/ncomms13071 (PMC5078730; doi:10.1038/ncomms13071)
Supplement: Supplementary Information — Supplementary Figures 1-8, Supplementary Tables 1-2, Supplementary Notes 1-4 and Supplementary References [file ncomms13071-s1.pdf]

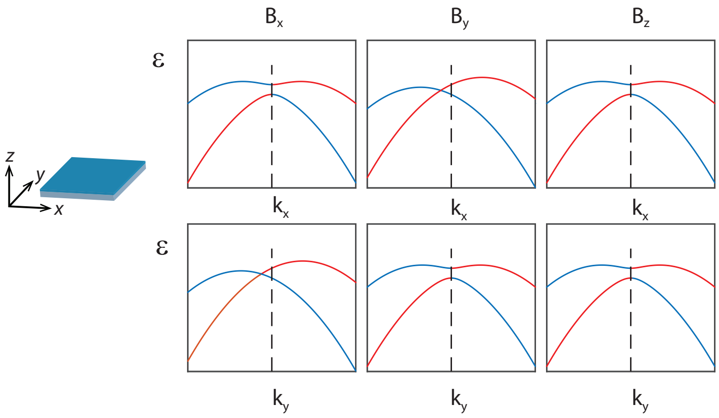

Supplementary Figure 1: **Two-dimensional Rashba-gas spectra under different magnetic field orientations.**

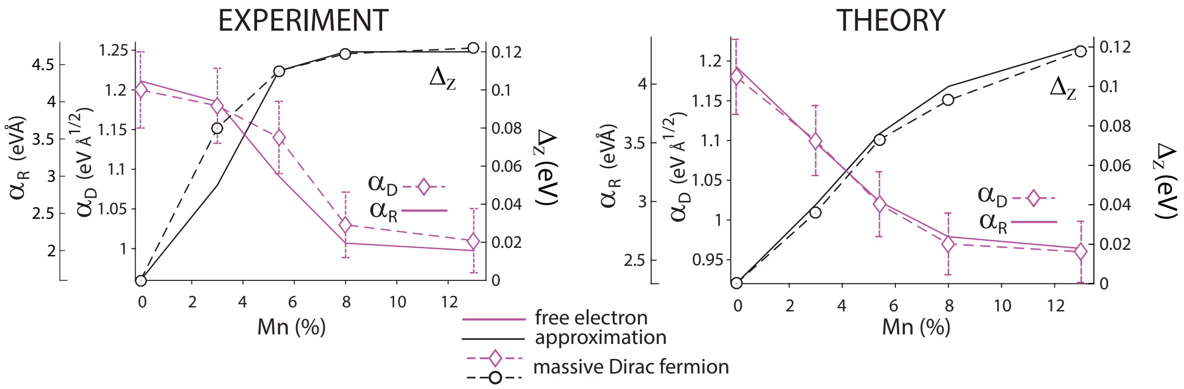

Supplementary Figure 2: **A comparison in fitting the Rashba strength parameters** α_R**,** α_D **and Zeeman gap** Δ_Z between experimental ARPES data (left) and multiple scattering theory (right) for the free electron approximation approximation (bold lines) and massive Dirac fermion model (dashed lines). The α_D error bars were obtained from varying the fit parameters.

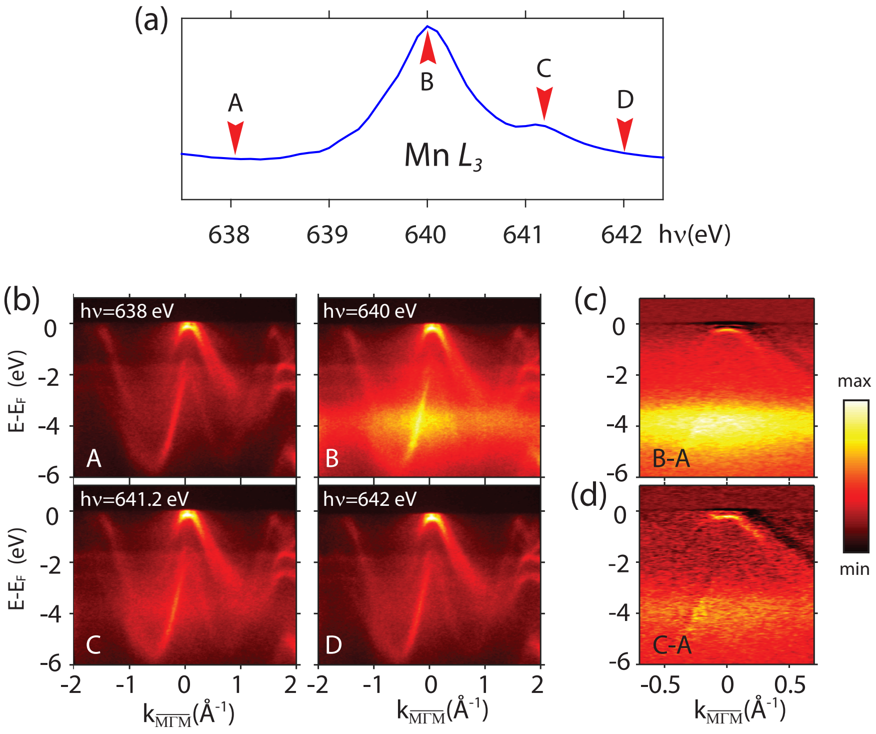

Supplementary Figure 3: **Resonant ARPES.** **(a)** X-ray absorption data at the L_3 absorption edge, the red arrows indicate the pre-resonance (A), resonance (B) and post-resonance (C,D) energies. **(b)** Series of ARPES spectra for the photon energies indicated in **(a)**. **(c)** Difference of the resonance spectrum B and the pre-resonance spectrum A; **(d)** difference between spectra C and A. The differential spectra visualize the dispersionless impurity band around 4 eV binding energy in **(c)** and the hybridization of the bulk Rashba-split bands of the α-GeTe host with Mn.

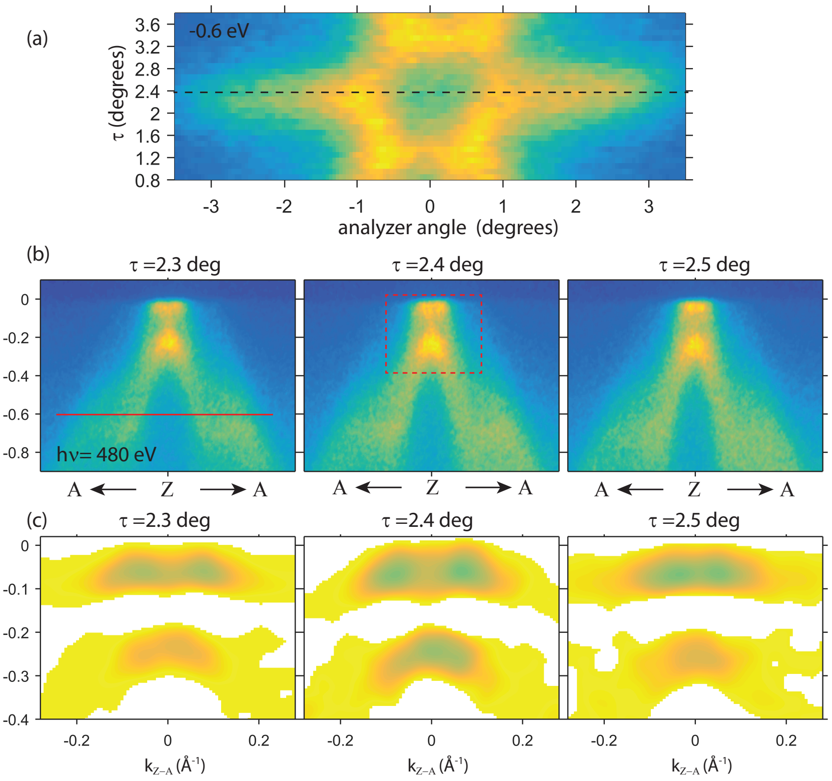

Supplementary Figure 4: **Sample alignment along** A-Z-A **direction.** **(a)** Constant binding energy cut at 0.6 eV binding energy for $Ge_{1-x}Mn_xTe$ x_{Mn}=5.4%, with corresponding raw ARPES band maps in **(b)** for three τ settings. The red line indicates where the bands intersect the constant binding energy cut in **(a)**. **(c)** Second derivative plots from red rectangle area in **(b)** shows higher gap openings around the former Dirac point at $\overline{\Gamma}$, indicating that τ=2.4° aligns the sample along A-Z-A direction with minimum gap opening (the Zeeman gap).

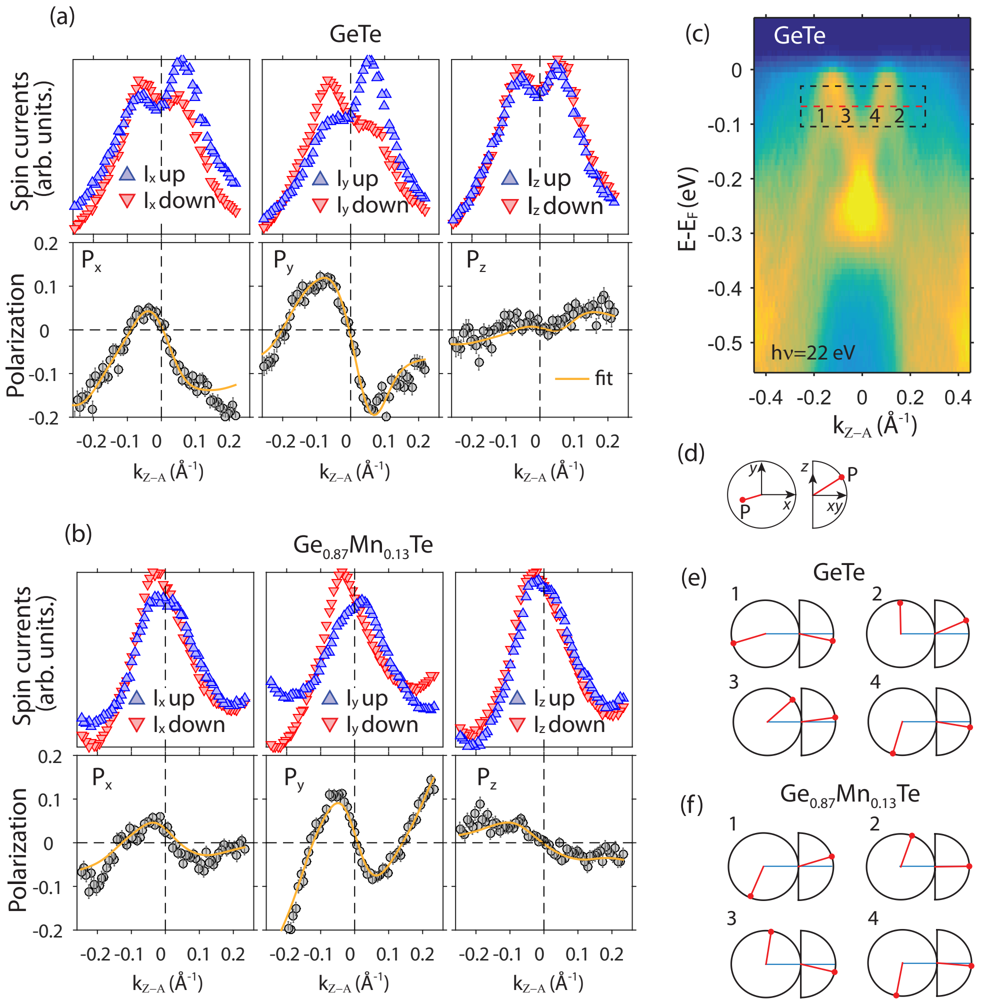

Supplementary Figure 5: **Comparison of spin components between α-GeTe(a) and $Ge_{0.87}Mn_{0.13}Te$ (b)** measured at E_F-80 meV along A-Z-A; recorded by the Mott detectors over the resolution-broadened energy range indicated by the dashed rectangle in **(c)**. The raw MDC intensities consist of four peaks: 1-2 are bulk derived and 3-4 are surface-derived Rashba-type states. **(d)** Definition of the local $\{xyz\}$ axes and polar representation of the 3D spin vectors (red ball-pointed arrows). **(e)** 3D spin vectors of the corresponding four bands for α-GeTe and **(f)** $Ge_{0.87}Mn_{0.13}Te$, confirming the equivalent in-plane spin-texture between the two.

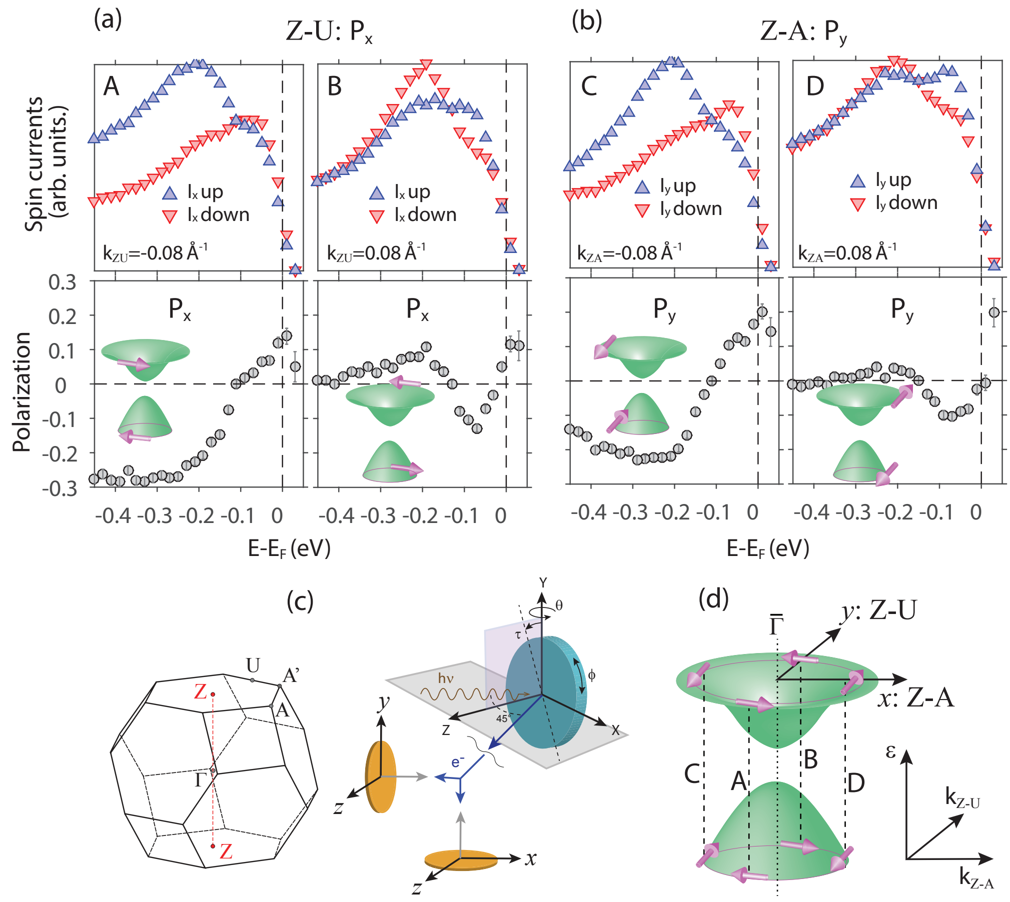

Supplementary Figure 6: **$Ge_{0.87}Mn_{0.13}Te$ bulk state in-plane spin texture above and below the Zeeman gap**, visualized by means of energy-resolved **(a)** P_x and **(b)** P_y polarizations for finite momenta $\pm 0.1 Å^{-1}$, labeled A-D. **(c)** Experimental geometry of the SARPES experiment with two orthogonal Mott detectors. **(d)** SARPES summary of P_x (P_y) polarizations measured along U-Z-U (A-Z-A) directions for the finite momenta A-D.

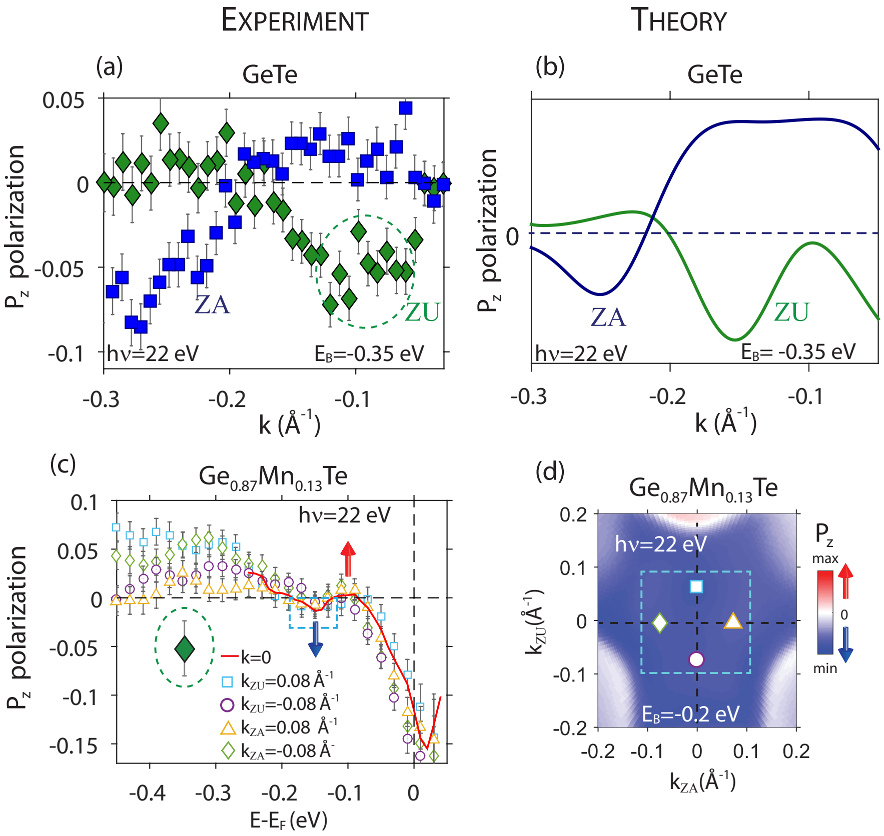

Supplementary Figure 7: α**-GeTe versus Ge$_{0.87}$Mn$_{0.13}$Te out-of-plane spin texture.** **(a)** MDC-resolved P_z-polarization measured at E_F-0.35 eV along Z-A and Z-U directions for α-GeTe(111)and; **(b)** the corresponding theoretical photoemission calculations. **(c)** EDC-resolved P_z for finite momenta along A-Z-A and U-Z-U. The red line indicate data measured in normal emission, also used in Fig. 4e of main text. **(d)** Calculated out-of-plane spin polarization in the photoemission final states at E_F-0.2 eV, overlaid with markers inside blue dashed frame indicating the finite momenta where the EDC-resolved P_z were measured in panel **(c)**. As a whole data in **(c)** account for smeared P_z due to ferromagnetic reorientation of the spins with characteristic spin wiggle across the Zeeman gap, being the primary signature of the Ge$_{0.87}$Mn$_{0.13}$Te spin reorientation (blue dashed frame in **(c)**. To give evidence on highly warped α-GeTe P_z spin texture, green dashed circle in panel **(c)** indicate where the α-GeTe P_z-data would be expected without the spin reorientation in Ge$_{0.87}$Mn$_{0.13}$Te.

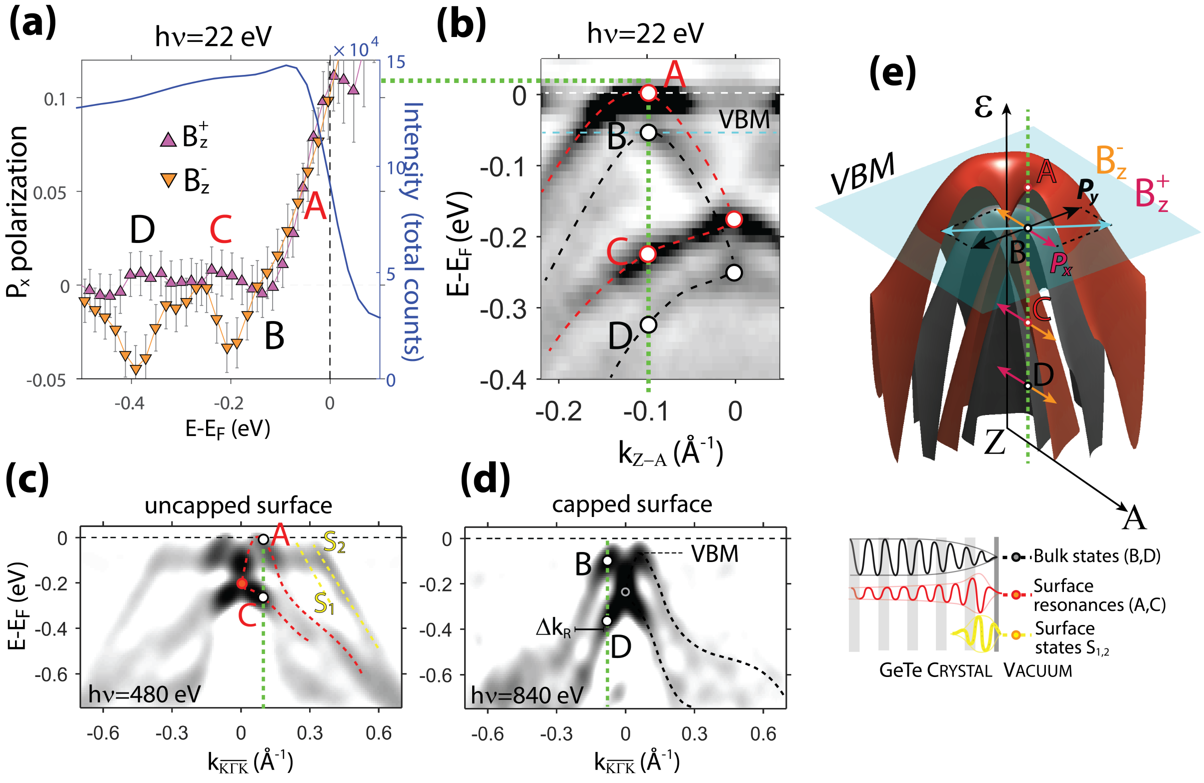

Supplementary Figure 8: **In-plane spin texture switching under magnetic field.** **(a)** $Ge_{0.87}Mn_{0.13}Te$ B-field control of the radial spin-polarization P_x for k= 0.1 $Å^{-1}$. **(b)** α-GeTe(111) second derivative ARPES band map measured at hν=22 eV from uncapped samples. **(c)** ARPES band maps measured at hν=480 eV from uncapped and **(d)** from capped film measured at hν=840 eV. The spectral features A, B, C, D in **(a)** are related to α-GeTe(111) ARPES data in **(b-d)**, the anatomy of the surface-resonance A,C (red color) and bulk bands B,D (black color) is sketched in **(e)** with a cartoon of their surface localization. The valence band maximum (VBM), situated around 60 meV below E_F, is marked by bulk band B. Its canted in-plane $P_{x,y}$ spin polarization is denoted with blue arrows in **(e)**.

Supplementary Table 1: ARPES Rashba-Zeeman-gas best-fit parameters.

Mn (%)	α_R (eVÅ)	Δ_Z (eV)	E_0 (eV)	m^*
0	4.3	0	-0.19	-0.15
3	4.0	0.05	-0.185	-0.15
5.4	3.0	0.11	-0.155	-0.19
8	2.1	0.12	-0.125	-0.30
13	2.0	0.12	-0.120	-0.32

Supplementary Table 2: ARPES Dirac-Rashba best-fit parameters.

Mn (%)	α_D (eVÅ$^{\frac{1}{2}}$)	E_Z (eV)	Δ_D (eV)	m_d ($\frac{1}{18\times10^{30}}$eV(Å/s)$^{-2}$)	v (10^5m/s)
0	1.20	0	0	0.21	4.60
3	1.18	0.080	0.28	0.22	4.50
5.4	1.14	0.110	0.43	0.49	3.70
8	1.03	0.119	0.44	0.60	3.50
13	1.01	0.122	0.45	0.60	3.45

Supplementary Note 1 Models for combined magnetic and spin-orbit order.

1.1 Effect of ferromagnetic order on a Rashba gas.

Here we describe the simplified two-dimensional Rashba-Zeeman-gas model discussed in the main text. We consider the momentum-dependent expectation values of the spin components interacting with ferromagnetic order defined by the unit vector $\hat{m} = (m_x, m_y, m_z)$ and expressed by means of the Zeeman splitting Δ_Z in energy . The Hamiltonian is given by

$$\mathcal{H} - E_0 = \frac{\hbar^2 k^2}{2m^*} + \frac{\Delta_Z}{2}\hat{m}\cdot\sigma + \alpha(k_x\sigma_y - k_y\sigma_x) - \mu, \tag{1}$$

where E_0 is the band bottom, m^* is the effective mass, α the Rashba parameter, Δ_Z the Zeeman gap, σ are the Pauli matrices and μ is the chemical potential. The energy eigenvalues are given by

$$E_{\pm}(k) = E_0 + \frac{\hbar^2 k^2}{2m^*} \pm \frac{1}{2}\sqrt{4\alpha\Delta_Z(k_x m_y + k_y m_x) + \alpha^2(k_x^2 + k_y^2) + \Delta_Z^2}. \tag{2}$$

Supplementary Figure 1 summarizes the resulting dispersions of the spin-polarized bands for different orientations of the magnetic field $\vec{B}$ along the $\{\mathrm{xyz}\}$ axes. Only for magnetization along the surface normal (z-axis) the degeneracy is lifted for both k_x and k_y momenta by opening the Zeeman gap.
For completeness Supplementary Table 1 summarizes all parameters used in the simplified Rashba-Zeeman-gas fit of the experimental data in Fig. 3 of the main text.

1.2 Dirac fermion with Rashba-like splitting and magnetic order.

It has been realized some time ago that the band dispersion of IV-VI semiconductors don't follow the typical free-electron like parabolic dispersion and that the bands can be better reproduced with a massive Dirac fermion model. The basic band dispersion in this case can be described by

$$E(k) = E_0 - \sqrt{m_D^2 v^4 + \hbar^2 k^2 v^2}. \tag{3}$$

Here m_D is the Dirac mass and represents the curvature for small k-values, v is the band velocity and represents the steepness of the bands for larger k-values, and E_0 is a band offset.

To this model the Rashba-like spin splitting and Zeeman gap opening can be added in a perturbative manner whereby it should be realised that, in order to keep a constant momentum splitting as a function of binding energy, the Rashba-like term should be of lower order as the main dispersive term. In the following we only consider magnetic order along the surface normal. This leads to the following phenomenological expression

$$E_{\pm}(k) = E_0 - \sqrt{m_D^2 v^4 + \hbar^2 k^2 v^2 \pm \frac{1}{4}\sqrt{\Delta_D^4 + 16\alpha_D^4 k^2}} \tag{4}$$

Here α_D is the Rashba-like parameter in units of eVÅ$^{\frac{1}{2}}$. It should be noted that the obtained values can only indirectly be compared to the Rashba parameters (in eVÅ) which are obtained for parabolic band dispersions. The Zeeman gap E_Z is directly proportional to the Δ_D energy term according to

$$E_Z = \sqrt{m_D^2 v^4 + \frac{1}{4}\Delta_D^2} - \sqrt{m_D^2 v^4 - \frac{1}{4}\Delta_D^2} \qquad (5)$$

Using this model the quality of the fit in Fig. 3 of the main text improves especially for larger momentum values. It should be noted that in this model the unit of the Rashba-like parameter α_D changes and therefore can no longer be directly compared to free electron approximation model discussed in the main text. The fitted results from both models are displayed in Supplementary Figure 2 for both ARPES data and multiple scattering theory. Both α_R and α_D show a similar dependency on Mn doping and also the Zeeman gap shows identical values for both models. This further reinforces the validity of the used models. The error bars for α_R ($\pm 7\%$, see Fig.3 of main text) and α_D ($\pm 4\%$) were obtained from varying the fit parameters (α_R and m^* for the free electron approximation; and α_D, m_d and v for the massive fermion model). By incorporating third, and higher order corrections in k the model can be further refined. However, in this case it is preferential to directly refer to the *ab-initio* calculations based on multiple scattering theory seen in Fig. 3. Finally, Supplementary Table 2 summarize the obtained fit parameters using the expanded massive Dirac fermion model.

Supplementary Note 2 Resonant ARPES across the Mn L_3 edge.

Here we illustrate the use of resonant photoemission to reveal the changes in the electronic structure of GeTe caused by the Mn doping. In this type of experiment one resonantly enhances the photoemission signal from certain elements by tuning the photon energy onto the corresponding peaks in the X-ray absorption spectrum (XAS). This yields elemental and chemical state resolution of the valence states (*1, 2*). In Fig. 2 of the main text, we could see that tuning the photon energy onto the main peak of the Mn L-edge XAS resonantly enhanced the Mn $3d$ related angle-integrated photoemission signal through the whole width of the valence band. We will now relate this resonant enhancement to particular bands resolved in electron momentum.
Supplementary Figure 3a reproduces the experimental Mn L-edge XAS from Fig. 2 of the main text. Supplementary Figure 3b shows ARPES data acquired at the photon energies indicated in Supplementary Figure 3a. Tuning the photon energy onto the main absorption peak at 640 eV unleashes the spectral weight directly connected with the Mn $3d$ states. The corresponding band structure image B of Supplementary Figure 3 differs from the pre-edge one (A) in two aspects:

- A Mn-derived impurity state appears at a binding energy of 4 eV. The absence of its k-dispersion reflects the random impurity character of this state. The enhanced energy broadening indicates a certain randomness of the local chemical environments of Mn atoms in the GeTe host.
- Whereas some of the dispersive GeTe host bands are insensitive to the resonant excitation, others significantly increase their intensity at the resonance, most evident in the region of the impurity band near the Z-point.

This difference reflects different strengths of the hybridization of these bands with the Mn states, depending on the particular character of the corresponding wavefunctions. The observed hybridization demonstrates integration of Mn states into the GeTe host states, consistent with the high solubility of Mn atoms in the GeTe lattice (*3, 4*). Of importance are two experimental observations:

- As seen in both difference spectra in Supplementary Figure 3c,d, the bands around the Zeeman gap are hybridized with Mn.

- The resonant image B in panel (b) and the difference image in (c), confirm the absence of any impurity states in the vicinity of E_F which might otherwise have interfered with the RZ-splitting in this region.

With a small increase of photon energy staying within the main absorption peak, the experimental band structure image C in Supplementary Figure 3a stays essentially the same as the resonant one except for a smaller Mn $3d$ intensity enhancement. The image D in Supplementary Figure 3b, taken above the resonance, is identical to the pre-resonant one (image A in Fig. 3b) apart from changes in k_z and a weak afterglow of the impurity state.

Supplementary Note 3 Experimental alignment of normal emission.

Due to the Dirac point singularity and the Zeeman gap opening in the former Dirac point in $Ge_{1-x}Mn_xTe$, accurate sample alignment with constant binding energy cuts is mandatory for all samples considered in Fig. 3c of main text. As an example, Supplementary Figure 4 shows the x_{Mn}=5.4% sample alignment along the A-Z-A direction, also seen in Fig. 3a of main text. Panel (a) shows that: (i) the correct manipulator τ setting is 2.4° along A-Z-A (see the experimental geometry in Supplementary Figure 6c); (ii) the required precision in manipulator settings is $0.1°$. Panel (b) shows raw ARPES band maps with the nearest τ settings around A-Z-A, together with their zoomed view panel (c).

Supplementary Note 4 Additional SARPES data.

4.1 SARPES 3D-vectorial peak-fitting analysis.

The COPHEE experimental station at the Swiss Light Source is a unique facility for SARPES experiments with a 3D Mott polarimeter (*5, 6*). Combined with an angle-resolving photoelectron spectrometer it produces complete data sets consisting of photoemission intensities as well as spin polarization curves for three orthogonal vector components. SARPES data in the main text show the populations of electrons with momentum along A-Z-A and U-Z-U having their spin parallel (up) or antiparallel (down) to the local momentum-dependent spin quantization axis. To ensure equivalent measurement conditions, data were taken by tilting the sample [the τ direction seen in Supplementary Figure 6c] with Z-A or Z-U oriented perpendicular to the scattering plane.

In a well established fitting routine (*7*) the photoemission spectrum is first dissected into individual peaks and background. Supplementary Figure 5(a,b) illustrate how the background subtracted total intensity momentum-distribution curves (MDC) are fitted with Voigt functions in both A-Z-A and U-Z-U directions. Supplementary Figure 5(c) shows ARPES data along A-Z-A measured at hν=22 eV, the dashed rectangle with red line in panel indicate the energy broadening of 60 meV and energy setting for α-GeTe and $Ge_{0.87}Mn_{0.13}Te$ SARPES measurements. The polarization curves are modeled until the best fit is reached by simultaneously fitting the MDC intensity and the polarizations P_x, P_y and P_z. The definition of the local $\{$xyz$\}$ axes and polar representation of the 3D spin vectors is seen in panel (d). A spin polarization vector is assigned to each peak-fit. Their lengths correspond to the degree of polarization, their in-plane angle is defined by the experimental geometry sketched in Supplementary Figure 6c. SARPES data were measured by rotating the sample azimuth ϕ such that Z-U or Z-A directions were aligned perpendicular to the scattering plane. In the sample coordinate system the x (y) axis was oriented along Z-U (Z-A) direction.

The orange lines inside the polarization panels P_x, P_y and P_z compare the output from the 3D-fit with

measured data summarized in Supplementary Figure 5e for α-GeTe(111) and Fig. 5f for $Ge_{0.87}Mn_{0.13}Te$, respectively. From the total intensities and the polarization data we generate the spin-resolved populations I_x, I_y and I_z along the three coordinate axes as a function of electron momenta. Consistently with the bulk Rashba initial states for α-GeTe (*8*), the $Ge_{0.87}Mn_{0.13}Te$ in-plane $P_{x,y}$ polarization feature canted spin helicity with nearly factor two higher P_y than P_x along A-Z-A. Thus, similar to α-GeTe, in $Ge_{0.87}Mn_{0.13}Te$ the in-plane spin texture is preserved. Their difference in P_z is to be attributed to the spin reorientation discussed in next section, combined with α-GeTeP_z-warping between the two equivalent Z-A and Z-A$'$ directions in the bulk Brillouin zone (Supplementary Figure 6c), as seen in Fig. 4a of main text.

4.2 Bulk states spin texture above and below the Zeeman gap.

To elucidate the spin texture above and below the Zeeman gap, and, to assess the overall in-plane $P_{x,y}$ spin texture of the Rashba-split bulk states, Supplementary Figure 6 visualize energy-resolved spin polarization for four finite momenta ($\approx \pm 0.1$ Å^{-1}) along U-Z-U and A-Z-A, denoted with A-D labels. For simplicity we ignore the canted spin and focus on maximum spin polarizations which are P_x along U-Z-U (Supplementary Figure 6a) and P_y along A-Z-A (Fig. 6b). Step-by-step the spin polarization in panels (a-b) above ($\approx$0.1 eV binding energy) and below the Zeeman gap ($\approx$0.3 eV binding energy) are indicated with violet arrows. All the spinors summarized in panel (d) show the opposite spin texture of the $Ge_{0.87}Mn_{0.13}Te$ bulk states just above and below the Zeeman gap, as also sketched in Fig. 1g of main text.

4.3 Out-of-plane spin texture comparison between α-GeTe and $Ge_{0.87}Mn_{0.13}Te$.

The band anisotropy between Z-U and Z-A induces a rather strong hexagonal warping which is responsible for the out-of-plane P_z spin polarization component (*9–11*). As mentioned in the main text, the symmetry breaking due to ferromagnetic order in the out-of-plane direction reorient the spins in the P_z-direction for $Ge_{1-x}Mn_xTe$. To substantiate this difference between α-GeTe and $Ge_{0.87}Mn_{0.13}Te$, Supplementary Figure 7 compare experimental data with one-step photoemission theory for each system. Our SARPES data in Supplementary Figure 7a measure out-of-plane spin polarization for α-GeTe along two inequivalent directions A-Z-A and U-Z-U, in agreement with the photoemission calculations shown in panel (b). Supplementary Figure 7c compares the $Ge_{0.87}Mn_{0.13}Te$ P_z-polarizations of the energy-resolved data A-D in Supplementary Figure 6 with α-GeTe. In all data the Pz-spinors exhibit the characteristic P_z-polarization modulation denoted by red line we discuss in the main text. This corroborates the smeared P_z-polarization below the Zeeman gap around 0.2 eV binding energy, in agreement with the photoemission calculations in panel (d). Finally, to give marked experimental evidence on the difference in P_z between α-GeTe and $Ge_{0.87}Mn_{0.13}Te$, dashed circle in Supplementary Figure 7a denote an energy-momentum locus of points totally absent in $Ge_{0.87}Mn_{0.13}Te$.

4.4 $Ge_{0.87}Mn_{0.13}Te$ in-plane spin texture under B-field switching.

The experimental setup at the Cassiopee beamline at Soleil Light Source, where the measurements with B-field switching were performed, allows to only investigate the out-of-plane and radial spin components of the Rashba-type spin texture. However, thanks to the peculiar canted spin texture in $Ge_{1-x}Mn_xTe$ discussed in Section IV.1, variations of the P_x radial spin component give insight in the change of the total in-plane spin component under B-field switching.

Supplementary Figure 8a shows spin-resolved EDCs for $Ge_{0.87}Mn_{0.13}Te$ for momenta around 0.1Å^{-1} while switching the B-field at ±700 Gauss. In order to understand the bulk spectral signatures in this data, Supplementary Figure 8 compares α-GeTe(111) ARPES band maps measured in the Z-point from uncapped samples along Z-A at hν=22 eV (panel b), hν=480 eV (panel c); and from capped samples at hν=840 eV (panel d). The comparison shows that ARPES in soft-X regime has greater bulk sensitivity (*12*), eventually accessing with higher photon energy of 840 eV the buried α-GeTe(111) layer under the cap where all the surface effects are quenched, revealing the α-GeTe(111) bulk electronic structure.

We note that ARPES data in Supplementary Figure 8 feature mixture of states with both bulk-derived (B and D) and surface character (A and C): A is at E_F, B is above the Dirac point, C and D are below the Dirac point. Displayed in red color are spectral features A and C which are surface resonance replica of the pure bulk states B and D denoted in black; and finally pure surface states $S_{1,2}$ are indicated in yellow. The anatomy of the bulk bands and their surface resonance replica is sketched in Supplementary Figure 8e.

As seen in Supplementary Figure 8a, in spin detection all the spectral features A–D also appear in $Ge_{0.87}Mn_{0.13}Te$. We note that the surface resonance A at E_F (also dominant at hν=480 eV on uncapped surface) does not change upon B-field switching. On the other hand spectral features B,C and D do indicate a change in the radial component: the bulk Rashba band B appear opposite to C and D. Furthermore, C and D consistently switch with the B-field because the spin polarization of the surface resonance replica C follows the bulk band D. The B-field switching in the P_x radial components is schematically depicted in Supplementary Figure 8e. Due to the experimental energy resolution (80-90 meV), the P_x spin reorientation in B is less evident because of the dominant contribution of A which does not switch under the considered B-fields. Yet the trend indicated by purple (B+) and orange (B-) arrows in Supplementary Figure 8e indicate a spin reorientation of the radial spin components for 0.1Å^{-1}.

Our experimental data only indirectly suggest that the tangential spin component P_y, indicated by black arrows in Supplementary Figure 8e, follows the P_x for the bands B,C,D with dominant bulk character. Their hybridization with pure bulk states is revealed by the ARPES band map from a capped surface in Supplementary Figure 8d by showing well resolved bulk Rashba bands with the indicated Δk_R around 0.1Å^{-1}.

As for the switching methodology under B-field, our results indicate that within the $Ge_{0.87}Mn_{0.13}Te$ surface electronic structure there are Rashba-type states with strong surface localization resilient to switch. On the other hand the states which hybridize with, or originate from bulk Rashba bands, do switch. Our conjecture is that a similar behavior will be also reflected in the E-field control of the spin texture. We note that due to the remanent magnetic field of the experimental station we cannot guarantee that the effective magnetic field M inside the sample was the same when switching the B-field between ±700 Gauss. This could be the reason for the difference in polarization amplitudes seen in Supplementary Figure 8a and Fig. 4d of main text.

Supplementary References

1. Molodtsov, S. L. *et al.* Angle-Resolved Resonant Photoemission as a Probe of Spatial Localization and Character of Electron States. *Phys. Rev. Lett.* **78**, 142–145 (1997).

2. Kobayashi, M. *et al.* Unveiling the impurity band induced ferromagnetism in the magnetic semicon-

ductor (Ga,Mn)As. *Phys. Rev. B* **89**, 205204 (2014).

3. Fukuma, Y. *et al.* Carrier-induced ferromagnetism in $Ge_{0.92}Mn_{0.08}Te$ epilayers with a Curie temperature up to 190 K. *Applied Physics Letters* **93**, 252502 (2008).

4. Przybylińska, H. *et al.* Magnetic-Field-Induced Ferroelectric Polarization Reversal in the Multiferroic $Ge_{1-x}Mn_xTe$ Semiconductor. *Phys. Rev. Lett.* **112**, 047202 (2014).

5. Hoesch, M. *et al.* Spin-polarized Fermi surface mapping. *J. Electron Spectrosc. Relat. Phenom.* **124**, 263–279 (2002).

6. Dil, J. H. Spin and angle resolved photoemission on non-magnetic low-dimensional systems. *J. Phys.: Condens. Matter* **21**, 403001 (2009).

7. Meier, F., Dil, J. H. & Osterwalder, J. Measuring spin polarization vectors in angle-resolved photoemission spectroscopy. *New J. Phys.* **11**, 125008 (2009).

8. Krempaský, J. *et al.* Giant Rashba splitting of bulk states in the ferroelectric αGeTe (111) semiconductor. *Preprint at https://arxiv.org/abs/1503.05004v1* (2015).

9. Di Sante, D., Barone, P., Bertacco, R. & Picozzi, S. Electric Control of the Giant Rashba Effect in Bulk GeTe. *Advanced Materials* **25**, 509–513 (2013).

10. Fu, L. Hexagonal Warping Effects in the Surface States of the Topological Insulator Bi_2Te_3. *Phys. Rev. Lett.* **103**, 266801 (2009).

11. Eremeev, S. V. *et al.* Atom-specific spin mapping and buried topological states in a homologous series of topological insulators. *Nat. Commun.* **3**, 103 (2012).

12. Fadley, C. S. Looking Deeper: Angle-Resolved Photoemission with Soft and Hard X-rays. *Synchrotron Radiation News* **25**, 26–31 (2012).
